# Delineating the Concerted Action of Virulence Attributes in *Candida albicans* Systemic Infection

**DOI:** 10.1111/jcmm.71353

**Published:** 2026-09-02

**Authors:** Olivia K. A. Paulin, Xiaoli Xu, Shu Chen Chong, Eve W. L. Chow, Li Mei Pang, Aaron D. Hernday, Jonathan P. Richardson, Yue Wang, Julian R. Naglik

**Affiliations:** ^1^ Centre for Host‐Microbiome Interactions, Faculty of Dentistry, Oral & Craniofacial Sciences King's College London London UK; ^2^ A*STAR Infectious Diseases Labs (A*STAR ID Labs), Agency for Science, Technology and Research (A*STAR) Singapore Singapore; ^3^ Department of Molecular Cell Biology, School of Natural Sciences University of California, Merced Merced California USA; ^4^ Temasek Life Sciences Laboratory Singapore Singapore

## 1 Introduction

*Candida albicans* is a World Health Organisation ‘critical priority’ pathogen [1], with systemic infections exhibiting a mortality rate of ~65% [2]. *C. albicans* possesses numerous virulence attributes to establish infection including hyphal formation, adhesion, toxin secretion and hydrolytic enzyme production [3]. Hyphal formation is regulated by proteins including Hgc1p, a G1‐cyclin critical for hyphal maintenance [4]; however, a *hgc1*Δ/Δ strain can still express hypha‐associated genes (e.g., *ALS3*, *ECE1*) [4]. Hyphal adhesion and invasion is mediated predominantly by Als3p through its interaction with host receptors such as E‐cadherin [3]. Hyphae secrete candidalysin (encoded by *ECE1*), a peptide toxin critical for host cell damage, mucosal translocation and induction of pro‐inflammatory immune responses [5]. *C. albicans* also secretes hydrolases including Sap2p, which degrade host proteins for nutrient acquisition and immune evasion [3].

Reverse genetics has identified virulence attributes important for *C. albicans* infection. However, while single knockout studies have provided key insights, the combined action of virulence genes during systemic infection remains poorly understood. Moreover, differing methodologies and genetic backgrounds limit cross‐study comparisons. Here, we generated a panel of mutant strains with additive deletions in a single genetic background, enabling systematic evaluation of individual and combined virulence factors during systemic *C. albicans* infection.

## 2 Results

### 2.1 Hyphal Growth and Candidalysin Are Critical for Controlling Early Fungal Burdens in the Brain

**FIGURE 1:**
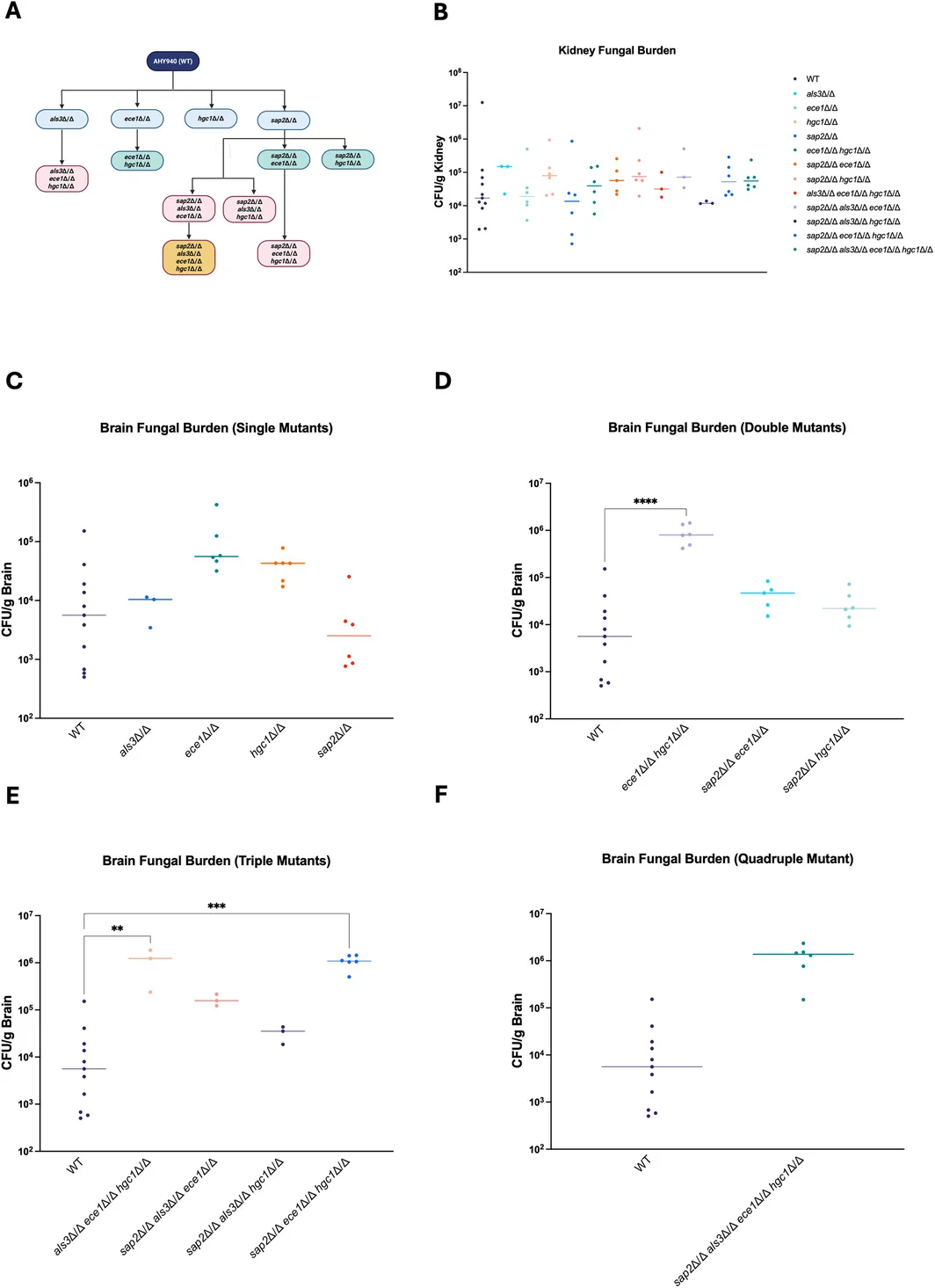
Early brain fungal burden is increased following deletion of *ECE1* and *HGC1*. (A) Systematic order of mutagenesis for each CRISPR deletion mutant constructed for this study. The background strain of AHY940 (WT) was used for all strains, with additive deletions being performed on mutated strains following CRISPR cassette recycling as previously described [6, 7]. Created with BioRender. (B–F) Fungal colony forming units (CFUs) in the kidney and brain were assessed following a 3‐day systemic infection. Mice were injected with 5 × 10^5^ *C. albicans* cells via the tail vein and the organs were harvested after 3 days (*n* = 3 mice/group per biological replicate. The following strains were assessed over two biological replicates (*n* = 6 mice total): *ece1*Δ/Δ, *hgc1*Δ/Δ, *sap2*Δ/Δ, *ece1*Δ/Δ *hgc1*Δ/Δ, *sap2*Δ/Δ *ece1*Δ/Δ, *sap2*Δ/Δ *hgc1*Δ/Δ, *sap2*Δ/Δ *ece1*Δ/Δ *hgc1*Δ/Δ and *sap2*Δ/Δ *als3*Δ/Δ *ece1*Δ/Δ *hgc1*Δ/Δ). (B) Kidney fungal burden in all mutants compared to WT. Brain fungal burden data have been separated into infection with (C) Single, (D) Double, (E) Triple and (F) Quadruple deletion mutants compared to WT. Data are presented as individual values obtained per mouse with median values depicted as a horizontal line. Statistical significance was calculated with one‐way ANOVA using a Dunnett's comparison test fixed to AHY940 (WT). ****p < 0.0001; ***p < 0.001; **p < 0.01.

The individual and combinatorial roles of *ALS3*, *ECE1*, *HGC1* and *SAP2* in *C. albicans* pathogenicity were assessed by constructing a panel of mutant strains with homozygous single, double, triple and quadruple gene deletions, as described previously [6] (Figure 1A). These mutants were investigated in a murine model of systemic infection.

Day‐3 analysis revealed no significant differences in kidney fungal burdens between any mutant strain and wild‐type (WT), suggesting that *ALS3*, *ECE1*, *HGC1* and *SAP2* are dispensable for controlling *C. albicans* growth in the kidney (Figure 1B). However, significant differences in median fungal burdens were observed in the brain, as follows: WT, 5620 colony forming units (CFU)/g tissue; *ece1*∆/∆, 55,900 CFU/g; *hgc1*∆/∆, 42,950 CFU/g; *ece1*∆/∆ *hgc1*∆/∆, 802,000 CFU/g; *als3*Δ/Δ *ece1*Δ/Δ *hgc1*Δ/Δ, 1,230,000 CFU/g; *sap2*Δ/Δ *ece1*Δ/Δ *hgc1*Δ/Δ, 1,058,000 CFU/g; *sap2*∆/∆ *als3*∆/∆ *ece1*∆/∆ *hgc1*∆/∆, 1,375,000 CFU/g. All other mutant strains exhibited brain CFUs similar to the WT strain (Figure 1C–F). Given that high fungal burdens correlated with the deletion of *HGC1* and *ECE1*, the data reveal that a combination of hypha formation/maintenance and candidalysin production promotes the control of fungal growth in the brain, with minimal roles for *ALS3* or *SAP2*.

### 2.2 Hyphal Formation, Candidalysin and Sap2p Secretion in Combination Promote Mortality

**FIGURE 2:**
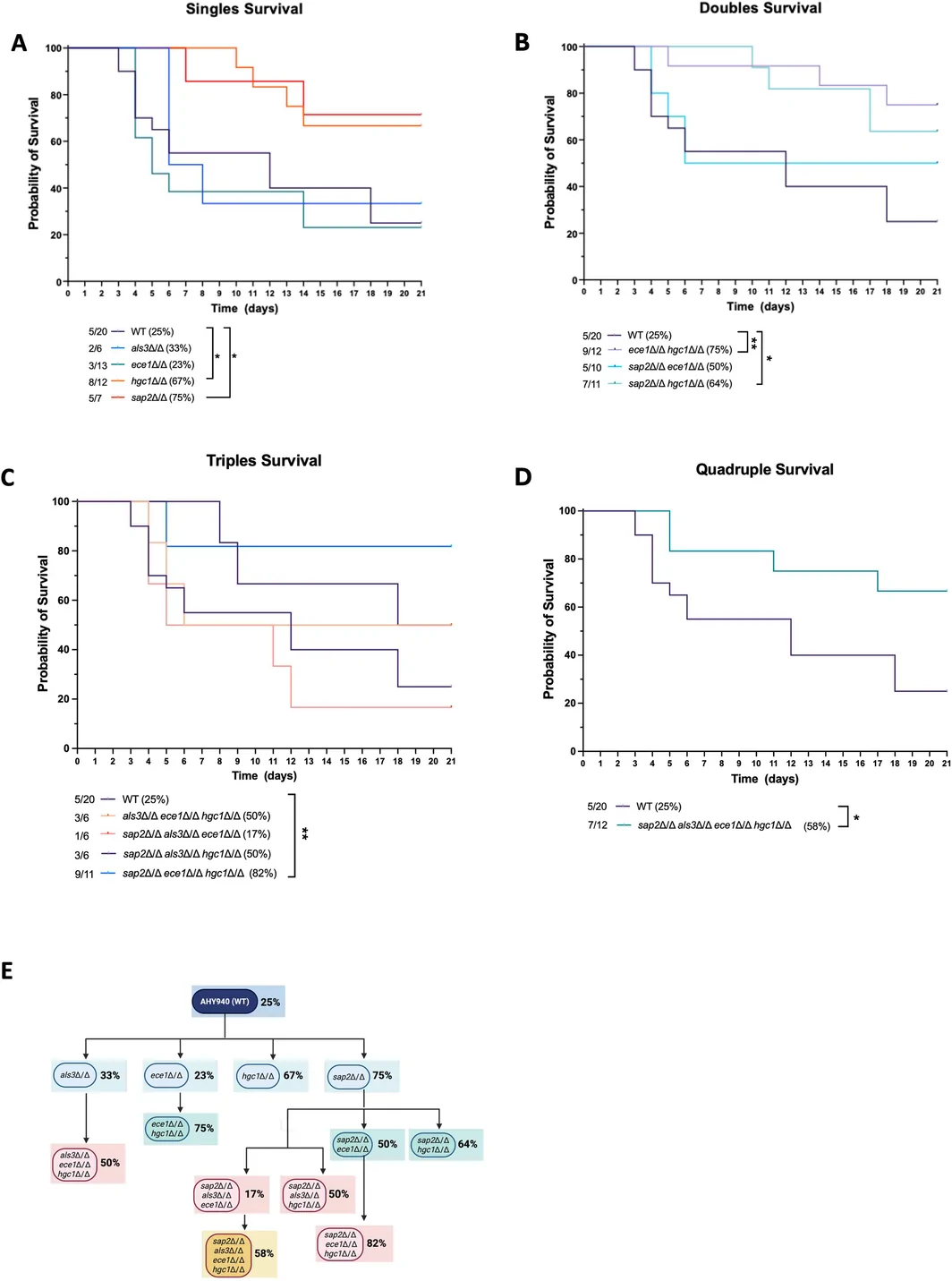
*HGC1*, *ECE1* and *SAP2* deletion promote survival in a murine model of systemic infection. (A–D) Mice were infected with 5 × 10^5^ *C. albicans* cells via the tail vein and were monitored for survival over 21 days (*n* = 4–6 mice/group per biological replicate, total mouse numbers are outlined in A–D panels. The following strains were assessed over two independent experiments: *Ece1*Δ/Δ, *hgc1*Δ/Δ, *ece1*Δ/Δ *hgc1*Δ/Δ, *sap2*Δ/Δ *ece1*Δ/Δ, *sap2*Δ/Δ *hgc1*Δ/Δ, *sap2*Δ/Δ *ece1*Δ/Δ *hgc1*Δ/Δ and *sap2*Δ/Δ *als3*Δ/Δ *ece1*Δ/Δ *hgc1*Δ/Δ). Data have been separated into infection with (A) Single, (B) Double, (C) Triple and (D) Quadruple deletion mutants compared to WT (AHY940). Data are presented as a pooled survival curve in which a vertical line depicts death. (E) Survival rate (%) across all investigated mutant strains in the systematic order of mutagenesis. (A–D) Statistical significance was calculated compared to AHY940 (WT) using a Kaplan–Meier survival statistical test. **p < 0.01; *p < 0.05.

To assess survival, mice were monitored for 3 weeks following systemic infection with *C. albicans* strains. Survival rates were as follows: WT, 25%; *als3*Δ/Δ, 33%; *ece1*Δ/Δ, 23%; *hgc1*Δ/Δ, 67%; *sap2*Δ/Δ, 75% (Figure 2A); *ece1*Δ/Δ *hgc1*Δ/Δ, 75%; *sap2*Δ/Δ *ece1*Δ/Δ, 50%; *sap2*Δ/Δ *hgc1*Δ/Δ, 64% (Figure 2B); *als3*Δ/Δ *ece1*Δ/Δ *hgc1*Δ/Δ, 50%; *sap2*Δ/Δ *als3*Δ/Δ *ece1*Δ/Δ, 17%; *sap2*Δ/Δ *als3*Δ/Δ *hgc1*Δ/Δ, 50%; *sap2*Δ/Δ *ece1*Δ/Δ *hgc1*Δ/Δ, 82% (Figure 2C); *sap2*Δ/Δ *als3*Δ/Δ *ece1*Δ/Δ *hgc1*Δ/Δ, 58% (Figure 2D). The single deletion mutants indicated that hypha formation and Sap2p were the strongest contributors to mortality. The multi‐deletion mutant data further revealed differing survival patterns following *ECE1* deletion depending on the genetic background, raising the hypothesis that the contribution of candidalysin to systemic virulence may be context‐dependent (Figure 2E). Als3p appeared to have a protective role in strains harbouring multiple deletions.

## 3 Discussion

Many studies have investigated the roles of *ALS3*, *ECE1*, *HGC1* and *SAP2* in *C. albicans* infections. However, comparisons between knockout strains are complicated by different genetic backgrounds and provide limited insight into combinatorial gene function during infection. To address this, we constructed single and combinatorial mutants in a single genetic background [6] and assessed virulence in a systemic murine model.

Although the kidney is generally considered the major target organ in murine systemic candidiasis, no clear role for each gene was observed in early kidney fungal control (day‐3). Interestingly, however, during brain infections, hyphal growth/maintenance (*HGC1*) and candidalysin (*ECE1*) were critical for controlling fungal burden. Complementing previously published datasets investigating single *ECE1* and *HGC1* deletions, deletion of *ECE1* significantly increased fungal burden compared to WT [8, 9], which was further exacerbated by concurrent *HGC1* deletion [8]. By comparison, deletion of *ALS3* or *SAP2* had minimal impact, suggesting limited roles for Als3p and Sap2p in brain infection. These findings likely reflect the combined effects of Hgc1p‐driven hyphal growth and candidalysin‐mediated immune activation, promoting protective responses via CARD9+ microglia and neutrophil recruitment [8]. Our findings therefore align with previously published work [8, 9].

Our Als3p data align with previous studies showing limited roles in endothelial adhesion and brain infection [10]. Although Sap2p has been implicated in brain entry [9], we observed only a modest reduction in fungal burdens with *sap2*Δ/Δ. This highlights how differences in experimental conditions and genetic background influence outcomes.

While day‐3 fungal burden reflects early innate immune control, it does not capture longer‐term disease progression. Therefore, 21‐day survival experiments were undertaken, revealing that hyphal growth/maintenance, Sap2p and candidalysin collectively contribute to virulence, with the greatest survival observed in mice infected with *C. albicans* deficient in Hgc1p, Sap2p and Ece1p. This complements previous datasets investigating the individual roles of *HGC1*, *SAP2* and *ECE1* in this model [4, 11, 12]. Notably, *ECE1* deletion alone resulted in WT‐like survival whereas survival was greater when *ECE1* and *HGC1* were deleted in combination. The highest survival was observed following additional deletion of *SAP2* (*sap2*Δ/Δ *ece1*Δ/Δ *hgc1*Δ/Δ). Conversely, mice infected with *sap2*Δ/Δ *ece1*Δ/Δ exhibited lower survival rates than *sap2*Δ/Δ‐infected mice (Figure 2E). Although these comparisons should be interpreted cautiously given the group sizes, they raise the hypothesis that the contribution of candidalysin to systemic disease may depend on the broader virulence and morphological context of the strain. One possibility is that loss of candidalysin in hypha‐producing strains reduces protective host responses including NLRP3 inflammasome [13] and adaptive Th17 responses [14], culminating in reduced inflammation and fungal clearance. Dedicated studies will be required to test this hypothesis. Interestingly, across all multi‐deletion mutants, *ALS3* deletion negatively impacted survival, suggesting a potential role of Als3p in inducing protective systemic host responses, possibly via NLRP3 inflammasome activation [15] or receptor engagement. This highlights that multiple gene deletions per strain would likely have profound impacts on host‐responses, which remains an important area for future enquiry.

The fungal burden data therefore indicate that hyphal growth and candidalysin production are associated with early restriction of fungal growth in the brain, whereas their role in the long‐term systemic outcome is contrasting, as these factors, alongside Sap2p, can also promote virulence. This highlights a previously reported uncoupling between early fungal burden and disease outcome [16, 17]. Fungal burden does not necessarily reflect strain pathogenicity, as host‐mediated tissue damage and subsequent organ dysfunction can also determine disease severity [16, 17]. One possible explanation is that fungal virulence attributes simultaneously promote protective immune responses that restrict fungal growth and host damage that contributes to mortality. Further studies investigating host responses to this mutant panel will be required to test this hypothesis.

In summary, this study demonstrates that hyphal growth and candidalysin production are critical for early control of brain infection, likely through induction of protective innate immunity. However, despite this, longer‐term survival studies using single deletion mutants revealed that hyphal formation and Sap2p were the key drivers of mortality, whereas candidalysin and Als3p play lesser roles, highlighting that fungal burden is not always reflective of virulence. Importantly, multi‐deletion mutant studies suggested candidalysin may play a dual role in virulence, in which *ECE1* deletion may be protective in the *hgc1*Δ/Δ background (yeast), yet detrimental in the *sap2*Δ/Δ background (hyphal). This may indicate a specialised, context‐dependent role for candidalysin depending on the fungal morphology, but further investigation is required. Overall, the most critical combination for *C. albicans* pathogenicity in the systemic model is hyphal growth, with concomitant candidalysin and Sap2p secretion, highlighting the importance of combinatorial virulence mechanisms in systemic *C. albicans* infection.

## 4 Materials and Methods

### 4.1 *Candida albicans* Strains and Growth Conditions

The *C. albicans* strains used in this study have been described previously [6] and were constructed using CRISPR‐Cas9 [7]. Strains were cultured at 30°C on yeast extract‐peptone‐dextrose (YPD) agar. Overnight cultures were grown for 16 h in YPD broth at 180 RPM, 30°C.

### 4.2 Murine Systemic *Candida albicans* Infection Model

Animal experiments were approved by the Institutional Animal Care and Use Committee of the Agency for Science, Technology and Research, Singapore. Six‐to‐seven‐week‐old immunocompetent, female C57BL/6 mice were injected intravenously via the tail vein with 5 × 10^5^ cells. Mice were sacrificed on day‐3 for organ fungal burden analysis, and remaining mice were monitored daily over 21 days for survival. Moribund mice or those ≤ 80% of their initial weight were humanely euthanised.

### 4.3 Organ Fungal Burden Analysis

At day‐3 post‐infection, mice were sacrificed for fungal burden quantification in the brain and kidney. Organs were harvested and homogenised and serial dilutions were plated onto YPD agar plates in triplicate.

## Author Contributions

**Olivia K. A. Paulin:** investigation, writing – original draft, methodology, writing – review and editing, formal analysis, conceptualization, validation, data curation. **Shu Chen Chong:** investigation, methodology. **Xiaoli Xu:** methodology, investigation. **Aaron D. Hernday:** conceptualization, methodology, writing – review and editing, supervision. **Julian R. Naglik:** supervision, funding acquisition, writing – review and editing, conceptualization. **Yue Wang:** conceptualization, writing – review and editing, supervision. **Eve W. L. Chow:** investigation, methodology. **Li Mei Pang:** investigation, methodology, writing – review and editing. **Jonathan P. Richardson:** conceptualization, writing – review and editing, supervision.

## Funding

This work was supported by Wellcome Trust (Grant 214229_Z_18_Z) and Biotechnology and Biological Sciences Research Council (Grant UKRI717).

## Conflicts of Interest

The authors declare no conflicts of interest.

## Data Availability

The data that support the findings of this study are available from the corresponding authors upon reasonable request.
